# Cold-stress induced metabolomic and transcriptomic changes in leaves of three mango varieties with different cold tolerance

**DOI:** 10.1186/s12870-024-04983-z

**Published:** 2024-04-10

**Authors:** Yu Kong, Xianbin Hou, Zhenglu Liu, Yufeng Li

**Affiliations:** 1 Guangxi Key Laboratory of Biology for Mongo, Baise University, Baise, 533000 China; 2 College of Agriculture and Food Engineering, Baise University, Baise, 533000 China

**Keywords:** Amino acids and derivatives, Chilling stress, Flavonoid biosynthesis, ICE-CBF-COR, Phytohormone signaling, Terpenoid biosynthesis

## Abstract

**Background:**

Mango (*Mangifera indica* L.) is grown in Hainan, Guangdong, Yunnan, Sichuan, and Fujian provinces and Guanxi autonomous region of China. However, trees growing in these areas suffer severe cold stress during winter, which affects the yield. To this regard, data on global metabolome and transcriptome profiles of leaves are limited. Here, we used combined metabolome and transcriptome analyses of leaves of three mango cultivars with different cold stress tolerance, i.e. Jinhuang (J)—tolerant, Tainung (T) and Guiremang No. 82 (G)—susceptible, after 24 (LF), 48 (MF) and 72 (HF) hours of cold.

**Results:**

A total of 1,323 metabolites belonging to 12 compound classes were detected. Of these, amino acids and derivatives, nucleotides and derivatives, and lipids accumulated in higher quantities after cold stress exposure in the three cultivars. Notably, Jinhuang leaves showed increasing accumulation trends of flavonoids, terpenoids, lignans and coumarins, and alkaloids with exposure time. Among the phytohormones, jasmonic acid and abscisic acid levels decreased, while N6-isopentenyladenine increased with cold stress time. Transcriptome analysis led to the identification of 22,526 differentially expressed genes. Many genes enriched in photosynthesis, antenna proteins, flavonoid, terpenoid (di- and sesquiterpenoids) and alkaloid biosynthesis pathways were upregulated in Jihuang leaves. Moreover, expression changes related to phytohormones, MAPK (including calcium and H_2_O_2_), and the ICE-CBF-COR signalling cascade indicate involvement of these pathways in cold stress responses.

**Conclusion:**

Cold stress tolerance in mango leaves is associated with regulation of primary and secondary metabolite biosynthesis pathways. Jasmonic acid, abscisic acid, and cytokinins are potential regulators of cold stress responses in mango leaves.

**Supplementary Information:**

The online version contains supplementary material available at 10.1186/s12870-024-04983-z.

## Background

Mango (*Mangifera indica* L.) is one of the world's most important fruits and is grown in more than 100 countries in both tropical and subtropical latitudes, particularly in Asia. According to World Population Review (www.worldpopulationreview.com), China is the third largest producer of mangoes after India and Indonesia. The scale of China's mango import volume and value (as of May 2023) increased by 2.7 and 2.3 times, respectively, compared to the previous year (www.weihengag.com accessed on August 08, 2023). This indicates the increasing market size of China's mango industry. Major mango producing areas in China are Hainan, Guangdong, Yunnan, Sichuan and Fujian provinces and Guangxi autonomous region [1]. In these regions, mango trees suffer from severe cold stress throughout the winter season, affecting their typical growth and development [2]. In particular, when the temperature drops below 10–12 ℃, young plants begin to show cold stress symptoms on their leaves [3, 4]. Several studies have shown that cold can directly affect photosynthetic potential and energy production, chlorophyll content, carotenoid content, increase in reactive oxygen species (ROS), while amino acid content is significantly reduced [5–7]. In addition to these biochemical attributes, transcriptome level studies have shown that cold tolerant genotypes exhibit higher gene expression for phytohormone-related genes, including jasmonic acid (JA), brassinosteroids (BRs) and others, MAPK signaling, and secondary metabolite biosynthesis [6]. Leaves are the photosynthetic organs and several leaf morphological and biochemical traits have direct link with the fruit growth, development, and quality [8]. Cold-induced photoinhibition can affect fruit development [9]. Mango fruits exhibit pitting, rooting, darkening of lenticel, changes in fruit peel color, and pulp quality traits [5]. Cold stress can increase abortion of embryos [10] and change fruit firmness by modifying cell-wall related polymers [11]. The effects of cold stress during ripening can also impact the postharvest quality of mango fruits [12]. In fruit peels, cold stress had an effect on carotenoid and flavonoid biosynthesis, phenylpropanoid biosynthesis, and genes related to oxidative stress [13]. Another study highlighted the potential role of cyclic nucleotide-gated ion channels in cold stress through regulation of malondialdehyde levels [14]. Using mango transplants, a recent study unraveled the role of salicylic acid (SA) in protecting mango plants from chilling stress [15]. Though these studies provide information on the molecular aspect of cold stress tolerance and susceptibility in mango trees, the information on how the seedlings and young trees respond to cold stress is limited [7]. Considering the fact that mango seedlings and young plants in the growing areas in China mentioned above are affected by cold stress, it is critical to understand the mechanisms of tolerance and susceptibility to cold stress in indigenous cultivars. This knowledge is essential to develop and provide potential strategies to protect mango seedlings from cold stress. Moreover, such knowledge is important for improving cold stress tolerance of existing cultivars.

Omics studies in other plants have revealed the involvement of multiple pathways in protecting plant leaves from cold injury. For example, a combined metabolome and transcriptome study in Cucurbita maxima showed that genes related to phytohormone signaling, carbohydrate metabolism, amino acid metabolism, phenylpropanoid pathway are highly expressed in the cold tolerant genotype [16]. Whereas in Capsicum annum, metabolome analysis revealed differential accumulation of phytohormones, polyamines and osmolytes along with increased expression of genes related to calcium, MAPK, phytohormone and reactive oxygen species (ROS) signaling [17]. A similar response has also been reported for tree species, e.g., apple [18], olive [19], rubber plant [20], *Populus tomentosa* [21], mulberry [22], and others. Research on the tree species that cope well with cold stress, such as Moso bamboo (Phyllostachys edulis), has revealed 10 to 1000 times higher levels of stress-related metabolites, i.e. glutathione, trehalose, and ascorbic acid. These changes are accompanied by a cold stress response transcriptome network consisting of genes involved in the above-mentioned pathways [23]. A large body of research on the model plant Arabidopsis and other crop plants has shown that when plants experience cold stress, they sense it through the ICE-CBF-COR pathway. In addition, Ca^2+^ acts as an important second messenger where its concentration in the cytosol changes rapidly. Studies have also shown the links between the ICE-CBF-COR pathway and Ca^2+^ signals. However, not only Ca^2+^ but also ROS act as signals in cold stressed plants [24]. The CBFs directly bind to COR genes promoter and activate their expression [25]. Although the mechanism of cold signaling and tolerance in several plant species has been well elaborated, we still lack a complete understanding in mango seedlings. Moreover, with the changing climate, temperature fluctuation and variable cold tolerance ability of mango cultivars, continued efforts are needed to explore the key molecular signatures present in tolerant cultivars and useful for breeding cold tolerant mango cultivars.

In this study, we take advantage of recent developments in transcriptomic and metabolomic technologies to investigate the key differential changes in the transcriptome and metabolome in leaves of three mango cultivars when challenged with cold stress for 24, 48, and 72 h. By analyzing these datasets, we discuss the differential accumulation of flavonoids in the leaves of cold tolerant mango cultivars. By analyzing these datasets, we discuss that the differential accumulation of flavonoids (FLs), lignans and coumarins (LCs), alkaloids, and terpenoids and associated transcriptome signatures enable Jinhuang to be cold tolerant compared to two other cultivars. These results provide several candidate pathways, metabolites, and genes for future studies as well as improving cold stress tolerance of susceptible varieties.

## Methods

### Plant material and cold treatment

Three mango cultivars i.e. Tainung 1 (T), Jinhuang (J) and Guiremang No. 82 (G) were provided by Base Tiandong National Mango Germplasm Resource Nursery, China. No permission is required to work on this species. Voucher specimens are available in the genebank Base Tiandong National Mango Germplasm Resource Nursery, China under the numbers: ASX671FT, ASX681FT and ASX911FT, respectively. Official identification of the plant material was conducted by Prof Yufeng Li. Based on biochemical profiles, Jinhuang had been characterized as cold tolerant [3], whereas Tainung 1 and Guiremang No. 82 were characterized as having poor cold tolerance, such that Guiremang No. 82 had relatively better cold performance than Tainung 1 [4]. The potted seedlings of the three cultivars were two years old at the time of sampling. The pots contained soil from the nursery soil and were mixed with sand so that the soil to sand ratio was 4:1. Ten plants of each cultivar were transferred to low temperature chambers (Climatron) for 24 (LF), 48 (MF), and 72 h (HF) at 4 ℃. Ten control (CK) plants for each cultivar were maintained at 26 ℃ (Table 1). The experiment was laid out according to completely randomized design with three replicates. At the required time points given in Table 1, disease- and insect-free leaves for each cultivar were harvested from triplicate plants. For each plant in each replicate, three leaves were collected and immediately frozen in liquid nitrogen.

**Table 1:** Layout of the experiment for cold treatment

Cultivar	Cold Treatment Time			
	0 h (C)	24 h (LF)	48 h (MF)	72 h (HF)
Tainung 1 (T)	TC	TLF	TMF	THF
Jinhuang (J)	JC	JLF	JMF	JHF
Guiremang No. 82 (G)	GC	GLF	GMF	GHF

### Widely targeted metabolome analysis

The biological samples were vacuum freeze-dried in a lyophilizer (Scientz-100F) and ground to powder (30 Hz, 1.5 min) in a grinder (MM 400, Retsch). The powder was then weighed on an electronic balance (MS105DM) and 1200 L of -20 °C pre-cooled 70% methanolic aqueous internal standard extract was added. The contents were then vortexed six times (30 s each time), followed by centrifugation at 12000 rpm for 3 min. The supernatant was aspirated, and the sample was filtered through a microporous membrane (0.22 mm pore size) and stored in the injection vial for UPLC-MS/MS analysis. Prior to storage in the injection vial, the extracts of the three leaves for each replicate were combined and processed as one replicate. The UPLC conditions were set as reported earlier by [26]. For ESI-Q TRAP-MS/MS analysis, the source operation parameters were set as follows (Table 2).

**Table 2:** ESI-Q TRAP-MS/MS operation parameters

Source temperature	500°C
Ion spray voltage	
Positive ion mode	5500 V
Negative ion mode	4500 V
Ion source gas I	50 psi
Ion source gas II	60 psi
Curtain Gas	25 psi
Collision-activated dissociation	High

The QQQ scans were acquired as multiple reaction monitoring (MRM) experiments. Nitrogen was used as the collision gas to acquire the QQQ scans. The declustering potential and collision energy were optimized for individual MRM transitions. Each period was examined for a specific set of MRM transitions based on the metabolites eluted during that interval.

For statistical analyses, we first computed unsupervised principal component analysis (PCA), hierarchical cluster analysis (HCA), and Pearson correlation coefficient (PCC) in R (www.r-project.com) using base, ComplexHeatmap, and corrplot functions, respectively. Furthermore, we screened the differentially accumulated metabolites (DAMs) based on variable importance project (VIP) > 1 as well as log2 foldchange ≥ 1. Subsequently, the metabolites were annotated in the KEGG compound database [27] and then mapped the DAMs on KEGG pathways, and their significance was determined by hypergeometric test’s *p*-values.

### Transcriptome analysis of cold stressed mango leaves

Freeze-dried leaf samples were ground in liquid nitrogen and RNA of each of three leaves in respective replicates was extracted using RNA Extraction Kit (QIANGEN). The quality of RNA was first checked by agarose gel electrophoresis and then by Agilent 2100 Bioanalyzer. After quality checking, the three RNA samples for each replicate were combined. Next, mRNA was extracted, first-strand cDNA was synthesized, and double-strand cDNA was synthesized and purified. The purified dscDNA was end-repaired, A-tailed, sequencing adapters were attached, followed by fragment size selection using AMPure XP beads. Finally, cDNA libraries were obtained by PCR enrichment. Library quality was determined using Agilent 2100 and Q-PCR. The libraries were then sequenced on the Illumina platform.

The sequencing data was filtered using fastp, and sequencing error rate and GC content distribution were checked. The clean data was compared with the reference genome [28] using HISAT2. The gene expression was quantified as FPKM (Fragments Per Kilobase of transcripts per Million fragments mapped) using featureCounts [29]. The PCC and PCA were computed based on the gene expression data in R. DESeq2 [30] was used for differential gene expression analysis. The genes having log_2_ foldchange value of ≥ 1 and false discovery rate < 0.05 were considered differentially expressed genes (DEGs). Based on the required comparisons, Venn diagrams were generated using interactiVenn. The DEGs were annotated according to KEGG, GO, NR, Swiss-Prot, TrEMBL, and KOG. The DEGs were also mapped on to the KEGG pathways for enrichment analysis.

### Real-time PCR analysis

The qRT-PCR was run as previously described by Yao et al. [31] using 14 selected genes and the housekeeping gene beta-tubulin *TUBB* (NCBI ID: *OP047694*) [31]. Primers (Table S1) were designed using Primer3Plus software [32].

## Results

### Metabolome profile of cold-stress leaves of three mango varieties

#### Global changes in leaf metabolome of cold stress mango varieties on different time points

**Fig. 1:**
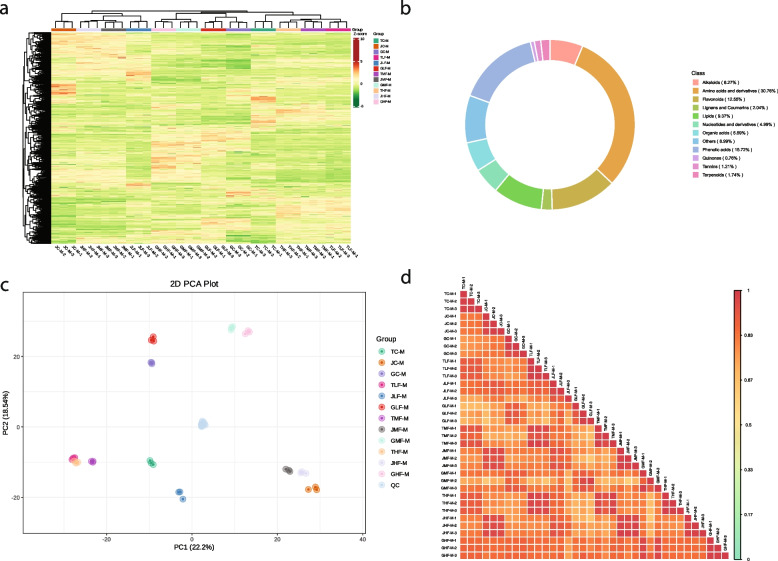
Widely targeted metabolome profile of cold-stress mango leaves of three varieties. **a** Heatmap all detected metabolites, **b**) pie-chart of the class of metabolites and % of compounds classified in each class, **c**) Principal component analysis, and **d**) Pearson correlation coefficient based on the relative compound intensities

UPLC-MS/MS analysis of 36 mango leaf samples identified 1,323 metabolites belonging to 12 compound classes (Fig. 1a). The highest percentage of compounds belonged to the class of amino acids and derivatives (AADs, 30.76%), followed by phenolic acids (PAs, 15.72%), and flavonoids (FLs, 12.55%) (Fig. 1b). The PCA plot based on relative intensities indicated that the replicates for each treatment were grouped together. It was also observed that GC, GLF, GMF, and GHF tended to group together. Similarly, the remaining three cultivars’ controls and treatments also tended to group together. These results indicate that the metabolomic profiles of the three cultivars are somewhat different (Fig. 1c). The PCC ranged from 0.61 to 1.0 with an average of 0.86 (Fig. 1d). These observations indicate the reliability of the sampling and replicates.

**Fig. 2:**
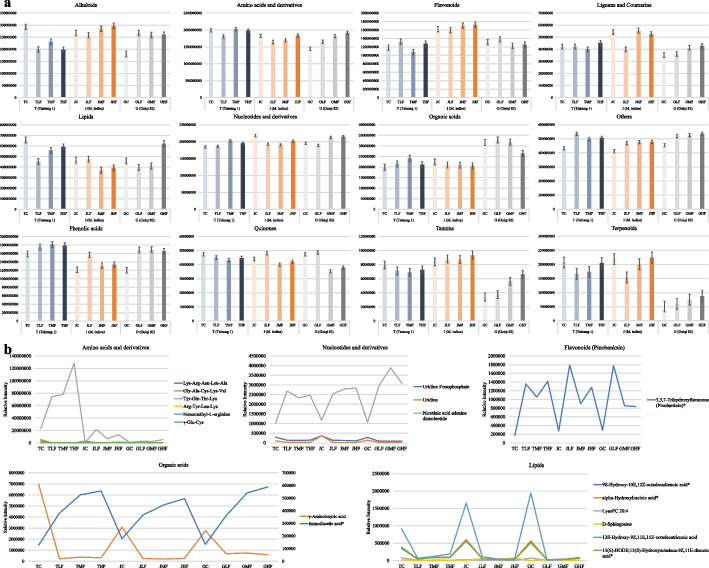
**a** Relative total content of different compound classes in three mango varieties at different time points after cold treatment. **b** Relative total content of 18 commonly accumulated metabolites in three mango varieties’ leaves at different time points after cold stress. The values used in the graphs are mean of three replicates

In particular, Jinhuang leaves with 0 h of cold exposure (JC) had higher lignans and coumarins (LCs), nucleotides and derivatives (NDs), and terpenoids compared to Tainung 1 and Guiremang No. 82 under the same conditions. This could be one of the reasons for the cold tolerance of Jinhuang compared to Tainung and Guiremang No. 82. In general, we found that the content of alkaloids, AADs, FLs, tannins and terpenoids increased from Jinhuang leaves with 24 h of cold exposure (JLF) to the ones exposed for 48 (JMF) and 72 h (JHF). This indicates that this genotype accumulates greater content of these compounds in leaves with the increase in time of cold stress. Whereas in Tainung 1 and Guiremang No. 82, alkaloids, FLs, LCs, and quinones either showed decreasing trend with cold stress exposure time or their trends were not consistent with stress time. We summed the metabolite intensities at each time point for each genotype, and Jinhuang had higher alkaloids, FLs, LCs, tannins, and terpenoids in leaves compared to Tainung 1 and Guiremang No. 82 (Fig. 2a). Therefore, it can be assumed that these compound classes could be a possible reason for better cold stress tolerance in Jinhuang leaves than in the other two cultivars. Finally, we identified the 18 major metabolites (AADs, lipids, NDs, organic acids (OAs), and FLs) that accumulated in all treatments and controls in three cultivars. In general, AADs increased after cold stress, indicating their universal role in cold stress responses. In particular, pinobanksin increased from 0 to 24 h, decreased at 48 h, and further increased at 72 h in Tainung 1 and Jinhuang leaves. Two OAs showed opposite accumulation trends. In contrast, lipids also showed variable trends (Fig. 2b).

Since Jinhuang is the cold tolerant variety, we identified 55 metabolites that showed an increasing trend in relation to cold stress exposure time. These compounds are classified as alkaloids, AADs, FLs, LCs, NDs, OAs, others (alcohol, ketone, saccharides, stilbene, and vitamins), PAs, and quinones, which were enriched in 38 KEGG pathways. However, not all of these metabolites showed the same trends in Tainung 1 (11 DAMs) and Guiremang No. 82 (12 DAMs) leaves. These metabolites with variation in accumulation trends were classified as AADs, OAs, and saccharides. On the contrary, we searched for the metabolites with a decreasing accumulation trend in cold-sensitive cultivars Tainung 1 and Guiremang No. 82 leaves and found 38 and 87 compounds, respectively. These were classified as alkaloids, AADs, FLs, NDs, OAs and PAs. Since these compounds did not show the same trend in Jinhuang leaves (except 6 compounds), they could be one of the possible reasons for the lower cold stress tolerance in Tainung 1 and Guiremang No. 82 leaves (Table S2).

The global metabolome profile highlights that the three cultivars’ leaves accumulate a range of metabolites in response to cold stress exposure.

#### Differential metabolome profiles of mango varieties’ leaves under the influence of cold stress

**Fig. 3:**
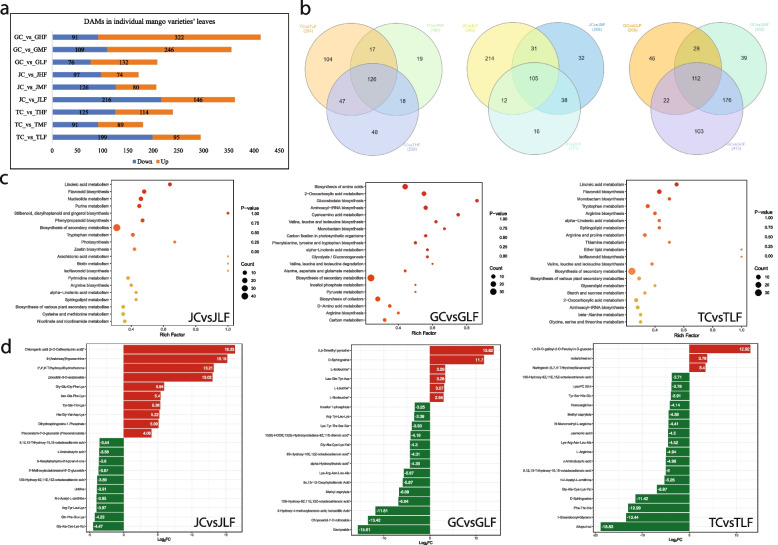
Differential metabolome profile of J, G, and T leaves stressed with cold. **a** Bar chart showing number of DAMs between control and cold treated leaves of three varieties. **b** Venn diagrams of DAMs accumulated in different treatment comparisons of each variety. **c**) Scatter plots of pathways to which the DAMs were enriched in mango leaves after 24 h of cold treatment compared to respective controls. **d** Top- and bottom DAMs in mango leaves after 24 h of cold treatment compared to respective controls

In the case of Tainung 1 leaves, there were 294, 180 and 239 DAMs in TLF, TMF and THF, respectively, compared to TC; 57 DAMs were commonly accumulated in the treatments studied. The other cultivars also had different numbers of DAMs compared to the respective controls. The highest number of DAMs were accumulated in GC vs. GHF (413), followed by JC vs. JLF (362), and GC vs. GMF (355). Both Tainung 1 and Jinhuang leaves had higher numbers of down-accumulated metabolites after cold stress in three treatments, whereas Guiremang No. 82 leaves had higher numbers of up-accumulated metabolites after cold treatment (Fig. 3a). By comparing the DAMs within cultivars, we found 126, 105 and 112 common DAMs in Tainung 1, Jinhuang and Guiremang No. 82 leaves, respectively (Fig. 3b).

KEGG pathway enrichment showed that DAMs (in JC vs JLF/JMF/JHF) were enriched in important metabolic pathways including FL biosynthesis, stilbenoid, diarylheptanoid, and gingerol biosynthesis, phenylpropanoid biosynthesis, tryptophan metabolism, purine biosynthesis, nucleotide metabolism, amino acid biosynthesis, arginine biosynthesis, linoleic acid metabolism, and others. In the case of Guiremang No. 82 and Tainung 1 leaves, DAMs were also enriched in glucosinolate biosynthesis, glycolysis/gluconeogenesis, and others (Fig. 3c; Figure S1). The top 10 up-accumulated metabolites in JLF compared to JC were PAs, NDs, ketones, FLs, and AADs. Interestingly, the down-accumulated DAMs in JHF were mainly AADs and lipids. Whereas in case of GLF, the most up-accumulated compounds were alkaloids, lipids and AADs and the most down-accumulated DAMs were terpenoids, FLs, lipids, OAs and AADs. Finally, in the case of TLF, the up-accumulated DAMs were PAs, FLs, and LCs, whereas the down-accumulated compounds were classified as NDs, AADs, lipids, and OAs (Fig. 3d; Figure S1).

Relatively higher number of metabolites classified as AADs, alkaloids, lipids, NDs and terpenoids were down accumulated after cold stress. On the other hand, most of the PAs and FLs were mostly up-regulated in all cold-stressed Jinhuang leaves. The 214 DAMs unique to JCvsJLF were mostly down-accumulated, i.e. alkaloids, AADs, terpenoids, and NDs. For FLs, LCs, lipids, and OAs, the number of down- and up-accumulated metabolites was mostly equal (Table S3). Among the JCvsJMF-specific DAMs, we observed that alkaloids (agmatine), LC (isofraxidin-7-O-glucoside), and PAs (except vanillic acid) were up-accumulated in JMF compared to JC, whereas all other metabolite classes were either down-accumulated or showed mixed accumulation patterns. Similar accumulation trends were observed for JCvsJHF specific DAMs (Table S3).

As for phytohormone-related metabolomic changes, we observed decreasing accumulation trends of ABA and JA. However, notably the JA content was higher in Jinhuang leaves than in Guiremang No. 82 and Tainung 1 leaves in the studied timepoints i.e., 0, 24, 48, and 72 h. The cytokinin (N6-isopentenyladenine) content increased in JLF and JHF compared to JC. In Tainung 1 leaves, however, its content increased in TLF but decreased in TMF and THF after cold stress. Only in Guiremang No. 82, its level increased with increasing cold stress time. In general, Tainung 1 leaves had a higher cytokinin content than Jinhuang leaves, while Guiremang No. 82 leaves had the lowest content. As for SA, its content increased in JLF and JHF compared to JC, while in Tainung 1 and Guiremang No. 82 leaves, then the content decreased after cold stress at all time points. Overall, Jinhuang leaves had a lower SA content than Tainung 1 and Guiremang No. 82 leaves (Table S3).

In general, we observed that cold induced a reduction of AADs (87 out of 129), lipids (55 out of 61), NDs (14 out of 17) and OAs (6 out of 17) in TLF, TMF and THF compared to TC. Notably, the content of phytohormones, i.e., jasmonic acid (JA) and abscisic acid (ABA), decreased in cold-treated TLF, TMF, and THF leaves compared to TC. In contrast, chilling induced accumulation of PAs (33 out of 46) and FLs (25 out of 33). Out of 104 unique DAMs in TCvsTLF, 86 were down-accumulated. Whereas, most of the unique DAMs in TCvsTMF (14 of 19) were up-accumulated in TMF compared to TC, and 20 of 48 unique DAMs in TCvsTHF were down-accumulated in THF compared to TC (Table S3). In the case of GLF, alkaloids, most AADs, saccharides, and PAs were up-accumulated (133 of 208). These DAMs continued to accumulate over longer time periods i.e., GMF and GHF. The lipids, NDs, OAs, ketones, and others were down-accumulated in GLF/GMF/GHF compared to GC. Interestingly, most of the FLs were not differentially accumulated in GLF and GMF and showed mixed accumulation trends in GHF. In contrast, a relatively higher number of DAMs (85 out of 104) were up-accumulated in response to prolonged cold stress, i.e., in GHF (Table S3).

Taken together, the metabolome comparison indicates that alkaloids, AADs, FLs, LCs, lipids, NDs, OAs, others (alcohols, ketones, saccharides, stilbenes, and vitamins), PAs, and quinones are cold stress-responsive metabolites in mango leaves. Among them, the increased accumulation of alkaloids, FLs, LCs, tannins and terpenoids in Jinhuang leaves indicates their strong role in cold stress tolerance.

### Transcriptome analysis of cold stress mango leaves

#### Global transcriptome profile of cold treated leaves of J, G, and T mango varieties

**Fig. 4:**
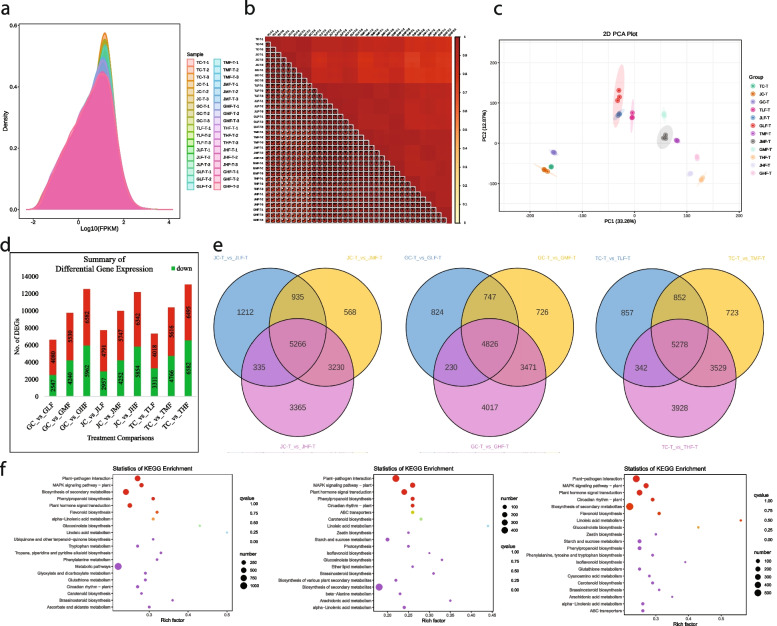
Transcriptome analysis of mango leaves treated with cold stress. **a** Expression (FPKM) density distribution map. **b** Pearson’s correlation and **c**) Principal component analysis based on FPKM values of the DEGs. **d** Summary of DEGs, **e**) Venn diagrams of DEGs in each variety, and **f**) scatter plots of pathways to which the DEGs were significantly enriched in JCvsJLF, GCvsGLF, and TCvsTLF

The sequencing of 36 libraries generated 49.49 million raw reads, which were processed to 48.36 million clean reads (261.15 Gb clean data). The average error rate, Q20, and GC content were 0.03%, 96.81%, and 42.21%, respectively (Table S4). Overall, 91% of the clean reads could be aligned to the reference genome. The FPKM distribution was lower in H (72 h of cold exposure) treatments and higher in C (0 h of cold exposure) for all varieties, indicating that cold stress significantly affects gene expression (Fig. 4a). The average PCC between varieties and treatments was 0.90, indicating the reliability of the replicates (Fig. 4b). The PCA showed that the treatments tended to group together (Fig. 4c). PC1 and PC2 could explain 33.26% and 12.87% of the variation, respectively.

#### Differential gene expression in cold stressed leaves of J, G, and T

The false discovery rate FDR < 0.05 and |log_2_ foldchange|≥ 1 resulted in the screening of 22,526 DEGs (Figure S2). In general, the number of DEGs increased with increasing cold stress (Fig. 4d). Within each cultivar, the number of common DEGs between treatment comparisons was 5266,4826, and 5278 in Jinhuang, Guiremang No. 82, and Tainung 1 leaves, respectively (Fig. 4e). DEGs in JCvsJLF, GCvsGLF, and TCvsTLF were significantly enriched in MAPK signalling, phytohormone signalling, beta-alanine metabolism, photosynthesis, flavonoid biosynthesis, alpha-linoleic acid biosynthesis, and several AAD and alkaloid-related pathways (Fig. 4f). In addition, with increasing stress time, a relatively higher number of DEGs were also enriched in sugar and starch biosynthesis related pathways (Figure S3).

#### Pathway-specific differential gene expression in cold stressed leaves of Jinhuang, Guiremang No. 82, and Tainung 1 leaves

Since the metabolome analysis indicated higher contents of alkaloids, FLs, LCs, tannins, terpenoids, and phytohormones in Jinhuang compared to Tainung 1 and Guiremang No. 82 leaves, therefore, we explored the expression changes in these pathways.

a Differential expression of genes related to photosynthesis

**Fig. 5:**
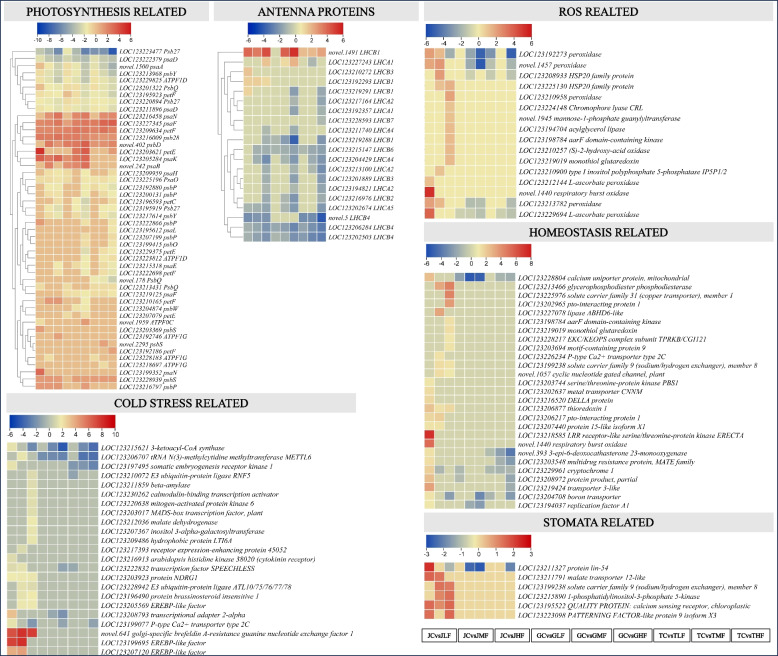
Heatmaps of DEGs enriched in pathways associated with photosynthesis and antenna proteins as well as GO terms associated cold stress, ROS, stomata, and homeostasis

Considering that the tissues under investigation are leaves, we first examined expression changes in genes enriched in photosynthesis and antenna protein pathways. In general, most of the photosystem (PS) I and II genes, viz, psaO, psb27, psbQ, psbY, psbW, psbP, psbO, psaL, psaH, psaF, psaE, psaD, psaA, ferredoxin (petF), plastocyanin (petE), cytochrome b6-f complex iron-sulfur subunit (petC), F-type H + -transporting ATPase-γ (ATP1G), and ATPF1D genes showed downregulation in response to cold stress. While others, i.e. psb28, psbS, psbD, psaN, psaK, a psaF (*LOC123227345*), petf (*LOC123209634*), petE (*LOC123203621*), were upregulated in response to cold stress. Notably, a psbS (*LOC123203369*), psbP (*LOC123216797* and *LOC123222866*), psaN (*LOC123199352*), petF (*LOC123210165*) were upregulated in Jinhuang leaves but not in Tainung 1 and Guiremang No. 82 leaves under the influence of cold stress. These genes could be a possible reason for the better photosynthetic potential in Jinhuang leaves. Apart from these changes, we also noticed that the number of downregulated transcripts associated with PSI and PSII were higher in Tainung 1 leaves followed by Guiremang No. 82 compared to Jinhuang leaves (Fig. 5). These changes, together with the up-regulation of light-harvesting complex II chlorophyll a/b binding protein 1 s and 3 in Jinhuang leaves after cold stress, possibly play important roles in its tolerance (Table S4).

b Differential expression of genes related to signalling

Next, we examined the expression trends of 683 transcripts enriched in phytohormone and MAPK signalling related genes under cold stress. The transcripts enriched in ethylene signalling showed mixed but similar expression in three genotypes, such that some transcripts with the same annotation were downregulated while others were upregulated. For auxin, one AUX1 (*LOC123228990*) was upregulated in Jinhuang (L, M, and H), Tainung 1, and Guiremang No. 82 (M and H) leaves, while another was upregulated only in Jinhuang leaves (L and M) and G (M). One TIR1 (*LOC123222571*) was upregulated in Jinhuang and Tainung 1 leaves but not differentially expressed in Guiremang No. 82 leaves, while two other TIRs were downregulated in G. Although AUX1/IAAs showed mixed regulation, some ARFs (*LOC123216984, LOC123206668, LOC123193211, LOC123226021, LOC123216813,* and *LOC123222603*) were specifically downregulated in Tainung 1 leaves and Guiremang No. 82 leaves but not differentially expressed in Jinhuang leaves. These changes suggest that Jinhuang leaves activates a relatively higher number of DEGs to transduce auxin signals downstream. Interestingly, a high number of DEGs (201) enriched in brassinosteroid (BR) signalling suggest their role in cold stress responses. The downregulation of more transcripts of CYCD3s and TCH4s in Tainung 1 and Guiremang No. 82 leaves compared to Jinhuang leaves with increasing cold stress time indicate that BR possibly protect the latter. We specifically explored the ABA and JA related expression changes because their content was affected (reduced) with cold stress as noted in metabolome analysis. ABA reduction possibly led to reduced expression of PYLs, but still several PYLs showed increase in expression after cold stress. PP2Cs showed opposite expression to PYLs. While two SnRK2s (*LOC123210266* and *LOC123193277*) and several ABFs had the same increasing expression trend in all genotypes with time of cold stress, others showed variable expressions in three genotypes. These changes confirm the metabolomic profile (Table S3). We observed large-scale changes in the expression of JA signalling related genes consistent with the reduction of JA in three genotypes after cold stress (Table S3). The reduction of JA is consistent with the downregulation of COI1s and upregulation of JAZs. Only one COI1 transcript was downregulated in Tainung 1 leaves after cold stress, which is consistent with the highest JA levels in Tainung 1 leaves at 0 h of cold exposure. Although we noted consistently increased expression of several MYC2s in three genotypes, the possible reason for better cold stress tolerance in Jinhuang leaves could be the upregulation of specific MYC2 transcripts, i.e., *LOC123229150* (in JLF), *novel.667* (in JMF and JHF), and *LOC123216793* and *LOC123195291* (in JHF). These are potential candidates for gene-specific characterization studies. The higher cytokinin content and increased expression of AHK2/3/4 transcripts (*LOC123216700* and *LOC123216913*) AHPs (*LOC123221649* specific to Jinhuang leaf samples). Similarly, only a limited number of ARR-B transcripts were downregulated in Jinhuang leaves, while specific transcripts i.e. *LOC123200785* were upregulated in JMF, JHF and GMF. The expression of these genes could possibly explain the involvement cytokinin signalling and downstream effects (Table S5).

In the case of MAPK signalling, the important branches of the pathway are related to abiotic stress are H_2_O_2_, reactive oxygen species (ROS), wounding, and stomata related. H_2_O_2_ is sensed by the serine/threonine protein OXI1. In the case of Tainung 1 and Guiremang No. 82 leaves, one OXI1 (*LOC123223725*) was differentially expressed only in GHF and THF. This could be a possible reason for reduced sensing of H_2_O_2_. Among the downstream genes of this pathway, i.e. ANP1, MKK4/5, MPK3/6 and WRKY22/29, one WRKY22 (*LOC123217366*) was upregulated in Tainung 1 and Guiremang No. 82 but not in Jinhuang leaves after 72 h of cold stress. Another WRKY22 (*LOC123196942*) was downregulated in JLF and GLF, indicating delayed senescence. Next, we observed that genes enriched in wound-related MAPK signalling were also differentially expressed. Among these, calmodulins (CaM) were upregulated with cold stress, but a notable observation was the downregulation of MKK3 (*LOC123196768* and *LOC123195593*) with cold stress. Further, respiratory burst oxidase (RbohD) accumulates the ROS burst. We observed that two RbohDs were consistently expressed in all genotypes, while one (*novel.1440*) was upregulated only in JLF. Interestingly, TLH activated five RbohDs, suggesting that it might had received more ROS compared to other genotypes. Finally, cold induces changes in stomatal development. For this, two genes, i.e., epidermal patterning factor 1/2 (EPF1/2) and LRR receptor-like serine/threonine protein kinase ERECTA (ER), together with MAPKKK YODA (YODA), MKK4/5, and MPK3/6, are expressed in sequence to alter the expression of TF SPEECHLESS. Among them, EPF1/2 s and several ERs were mostly downregulated with increase in stress timing in three genotypes. However, we noticed that two SPEECHLESS transcripts (*LOC123215969* and *LOC123222832*) had higher expression in Jinhuang than in Tainung 1 and Guiremang No. 82 leaves, while two were consistently downregulated in all genotypes. Taken together, these changes indicate that the expressions of OXI1, WRKY22s, CaMs, ER, and SPEECHLESS play important roles in signal transduction in Jinhuang leaves and enable it for cold stress tolerance (Table S5).

Since signalling directly affects homeostasis, stomata, cold stress, and ROS-related gene expression changes, we explored the related GO terms and found 467, 260, 362, and 270 DEGs, respectively. Among them, we found 27, six, 23, and 16 DEGs, respectively, that were upregulated in Jinhuang leaves at one or more time points. However, these genes were not differentially expressed or downregulated in Tainung 1 and Guiremang No. 82 leaves after cold stress (Fig. 5; Table S5). These genes belonged to 62 KO terms and are strong candidate genes for functional characterization of cold stress tolerance in mango.

**Fig. 6:**
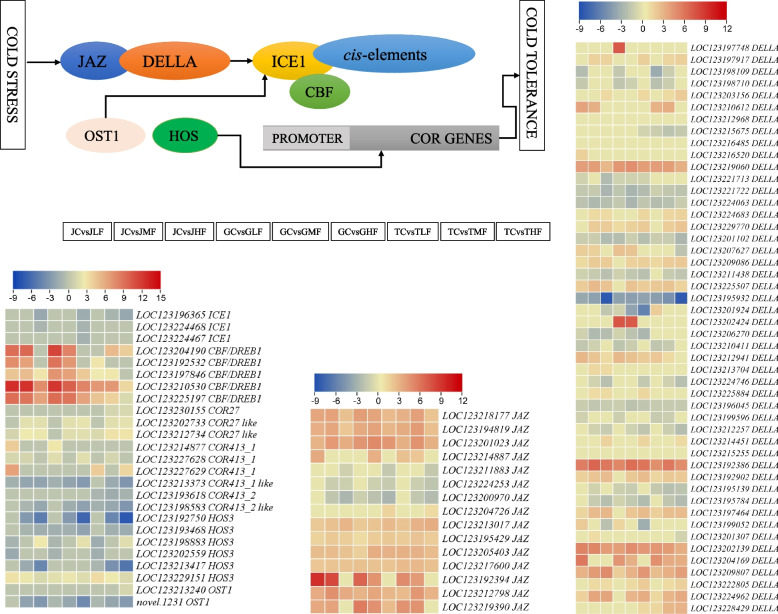
ICE-CBF-COR related differential gene expression in three mango genotypes’ leaves after cold stress treatment. The heatmaps represent log2 foldchange values

Since the metabolome showed an increased accumulation of FLs in response to cold stress, especially in Jinhuang leaves, we examined the expression changes of genes (85 transcripts associated with 16 KO terms) enriched in this pathway. Notably, 21 genes including flavonoid 3',5'-hydroxylases (F3′5'H), bifunctional dihydroflavonol 4-reductase/flavanone 4-reductase (DFR), shikimate O-hydroxycinnamoyltransferase (HCT), 5-O-(4-coumaroyl)-D-quinate 3'-monooxygenase (C3'H), flavonoid 3'-monooxygenase (F3'H), chalcone synthase (CHS), caffeoyl-CoA O-methyltransferase (CCoAMT), trans-cinnamate 4-monooxygenase (CA4H), and naringenin 3-dioxygenase (F3H), were highly expressed in one or more treatments in Jinhuang leaves. However, these genes were either downregulated or not differentially expressed in Tainung 1 and Guiremang No. 82 leaves (Fig. 6; Table S5). These expressions are consistent with the changes in FLs in the three genotypes. These results suggest that the differential flavonoid content is related to cold stress tolerance in the genotypes studied.

c Differential expression of genes related to amino acids and derivatives

Regarding AADs, 213 DEGs were enriched in the biosynthesis pathway of amino acids. Generally, the number of up-regulated transcripts increased with the time of cold stress in the three varieties with the highest in Jinhuang, followed by Tainung 1 and Guiremang No. 82 leaves. Notably, some genes such as glutamine synthetase (GS, *LOC123197930* and *LOC123205754*), threonine synthase (*LOC123211337*), enolase (*LOC123203890*), 3-deoxy-7-phosphoheptulonate synthase (*LOC123205490*), shikimate kinase (*LOC123202729*), pyruvate kinase (*LOC123197276*), ATP phosphoribosyltransferase (*LOC123213839*), serine O-acetyltransferase (*LOC123192433*) were up-regulated in Jinhuang leaves in at least one or more time points. Whereas, these genes were mostly not differentially expressed in Guiremang No. 82 leaves after cold stress. These observations confirm the relatively higher accumulation of DAMs in Jinhuang after cold stress, followed by Tainung 1 and Guiremang No. 82 leaves. Thus, confirming the differential metabolomic profiles of studied cultivars (Table S5).

d Differential expression of genes related to terpenoids

The metabolomic profiles also indicated an increased terpenoid content in the genotypes. However, Jinhuang leaves had higher contents than Tainung 1 and Guiremang No. 82 leaves, which is consistent with their cold tolerance potential. In total, 191 DEGs were enriched in terpenoid-related pathways, i.e. terpenoid backbone biosynthesis, mono-, di-, ubiquinone and other terpenoid quinone biosynthesis, sesquiterpenoid, triterpenoid biosynthesis, and stilbenoid, diarylheptanoid and gingerol biosynthesis pathways (Table S5). In the case of terpenoid backbone biosynthesis, we noticed trimethyltridecatetraene/dimethylnonatriene synthase, many beta-amyrin synthases, (-)-germacrene D synthases, an o-succinylbenzoate CoA ligase, many shikimate O-hydroxycinnamoyltransferases, a 5-O-(4-coumaroyl)-D-quinate 3'-monooxygenase, an ent-copalyl diphosphate synthase, several 4-coumarate–CoA ligases, tyrosine aminotransferases, caffeoyl-CoA O-methyltransferases, squalene monooxygenase, and a transcinnamate 4-monooxygenase were up-regulated in Jinhuang leaves after cold stress. In the case of Tainung 1 leaves, some of these were upregulated in TLF and TMF, but mostly downregulated or not differentially expressed in Guiremang No. 82 leaves after cold stress. This suggests that the biosynthesis of higher terpenoids in Jinhuang and Tainung 1 leaves may start from the terpenoid backbone biosynthesis pathway. DEGs related to monoterpenoid biosynthesis were mostly up-regulated in Tainung 1 and Guiremang No. 82 leaves after cold stress and either down-regulated (®-limonene synthase) or not differentially expressed in Jinhunag leaves after cold stress, except for two transcripts of ( +)-neomenthol dehydrogenase. In contrast, most of the diterpenoid-related DEGs were generally differentially expressed in all genotypes after cold stress, while some were up-regulated in Jinhuang leaves after cold stress. This suggests that in diterpenoids play a role in cold stress tolerance in Jinhuang leaves. The same trend was observed for sesquiterpenoid pathway genes. In particular, beta-amyrin synthase, (-)-germacrene D synthase, and squalene monooxygenase were upregulated in Jinhuang leaves, but were either downregulated or not differentially expressed in Tainung 1 and Guiremang No. 82 leaves. Thus, these observations confirm the metabolomic findings and suggest that terpenoids enable Jinhuang leaves to better tolerate cold stress.

e Differential expression of genes related to lipid biosynthesis/metabolism

Next, we explored the pathways related to lipid biosynthesis/metabolism, because in the case of Jinhuang leaves, we noted an increase in lipid content in JLF, which then decreased, but in Tainung 1 leaves, lipids increased with time. In the case of Guiremang No. 82 leaves, lipids increased only in GHF. A total of 315 DEGs were enriched in four lipid pathways, i.e. glycerophospholipid, glycerolipid, sphingolipid and ether lipid metabolism. Consistent with the DAM data, we found that an increasing number of DEGs were enriched in lipid metabolism in Jinhuang and Tainung 1 leaves. In particular, several phospholipase A1s, lysocardiolipin and lysophospholipid acyltransferases, beta-galactosidases, glycerol-3-phosphate O-acyltransferase, and an aldehyde dehydrogenase (NAD +) were upregulated in Jinhuang and Tainung 1 leaves after cold stress, but were either not differentially expressed or downregulated in Guiremang No. 82 leaves (Table S5). These expression changes are consistent with the observed accumulation of the metabolome.

f Differential expression of genes related to alkaloid biosynthesis

Next, two alkaloid biosynthesis-related pathways, i.e. tropane, piperdine and pyridine alkaloid biosynthesis and isoquinoline alkaloid biosynthesis, were enriched with 41 DEGs. Among these DEGs, three tropinone reductase I (*LOC123209012*, *novel.288*, and *LOC123193003*) and one tyrosine aminotransferase (*LOC12 3195583*) were upregulated only in Jinhuang and Tainung 1 leaves. Three chalcone synthases (*LOC123221036, LOC123221037,* and *LOC123222546*) and a primary amine oxidase (*LOC123213509*) were specifically upregulated in JLF (and/or JMF) after cold stress. Other transcripts were downregulated in all treatments of three genotypes. In general, the expression trends of DEGs in these pathways showed increasing trends in Jinhuang but not in Tainung 1 and Guiremang No. 82 leaves, which is consistent with the accumulation of alkaloids after cold stress.

g Differential expression of genes related to ICE-CBF-COR signalling cascade

Finally, we screened the genes based on the annotations related to ICE-CBF-COR signaling cascade pathway. Three inducers of CBF expression 1 (ICE1), five CBF/DREB1, three COR27, six COR413, 48 DELLA, six high expression of osmotically responsive gene 3 (HOS3), 16 JAZs, and two open stomata 1 (OST1) were differentially expressed in the three genotypes at the studied cold stress time points. The ICE1 showed downregulation when cold stress was extended to 72 h of cold stress in three genotypes. However, the number of downregulated transcripts was least in Tainung 1, followed by Jinhuang and Guiremang No. 82 leaves. The CBF/DREB1 transcripts were upregulated after cold stress in all genotypes. One COR27 (*LOC123230155*) was upregulated only in TMF and THF compared to TC, while (*LOC123212734*) showed consistent upregulation in all genotypes. However, those annotated as COR413_1-like, COR413_2 (specific to Tainung 1 leaves) and COR413_2-like (except in JLF and GLF) were downregulated. Notably, COR27 transcripts had higher FPKM values than COR413. Three, two and one COR413_1 transcripts were upregulated in JLF, GLF and TLF, respectively. DELLAs (GAI, GAI-like, GAI1-like, GAIP-B-like, SLR1-like) and JAZs showed variable expression. However, among DELLAs, we found genotype and treatment specific transcripts. In the case of HOS3, two transcripts (*LOC123192750* and *LOC123193468*) were downregulated in 48 and 72 h treated leaves of all genotypes. Among other HOS3 transcripts, *LOC123198883* was specifically upregulated in JHF, GHF, and THF, while *LOC123229151* was consistently expressed in all genotypes. OST1 (*novel.1231*) was downregulated, whereas *LOC123213240* was upregulated exclusively in THF. These observations suggest that ICE-CBF-COR signaling cascade genes are activated in response to cold stress in all genotypes, and CBF/DREB1 and COR413_1 might differentially regulate cold tolerance in Jinhuang, Guiremang No. 82, and Tainung 1 leaves.

To validate the gene expression profile obtained from the RNA-seq in this study, we selected 14 candidate genes and evaluate their transcript levels using the qRT-PCR method. As presented in Figure S4, all genes showed differential expression among time points. In addition, a high and positive correlation was obtained between qRT-PCR and RNA-seq, indicating that the gene expression measured in the RNA-seq study is reliable.

Taken together, the metabolomic and transcriptomic data clearly indicate that FLs, LCs, alkaloids, tannins, and terpenoids are key regulators of cold stress tolerance, and their higher levels under cold stress are associated with increased gene expression for DEGs enriched in these pathways. The transcriptome data also indicate that phytohormone, MAPK, and ICE-CBI-COR signaling pathways play significant roles in the differential cold response of the three genotypes.

## Discussion

Cold stress in mango plants at early age is devastating and can affect leaf morphology, which is an important organ for photosynthesis and transpiration. Limited knowledge on the metabolomic and transcriptomic changes in mango leaves with different cold stress tolerance potential motivated us to initiate this study. Upon exposure to cold stress, plants activate sophisticated molecular mechanisms that relay signals to different metabolic processes to enable plants to survive cold [33]. The changes in Ca^2+^ and ROS flux initiate signaling cascades that are part of plant-pathogen interaction and MAPK signaling plant pathways. The observation that CaM, MKK3, RbohD were differentially expressed clearly indicates cold-induced changes related to Ca^2+^ signaling [34]. Increased H2O2 production in response to cold stress is sensed by OXI1. The upregulation of OXI1, ANP1, MKK4/5, MPK3/6, and WRKY22/29 in T and G after 72 h of cold stress indicates that these genotypes have higher ROS in response to cold stress. On the other hand, the downregulation of WRKY22 in Jinhuang and Guiremang No. 82 leaves indicates that these genotypes can survive cold better than Tainung 1, which is consistent with a previous report that wrky22 Arabidopsis mutants exhibit delayed senescence [35]. Additionally, the upregulation of an RbohD (*novel.1440*), which have been implicated in rapid systemic signaling during multiple stresses [36]. The signals are then relayed to homeostasis-related genes. The up-regulation of 27 homeostasis-related genes and several MAPKs in Jinhuang leaves and the contrasting expressions in Guiremang No. 82 and Tainung 1 leaves clearly indicate that the former can better manage the influx of ROS (Table S4; Fig. 5). A similar mechanism has been reported in Arabidopsis [37]. MAPK signals also involve pathway for stomatal development, where signals are mediated by EPF1/2, ER, YODA, MKK4/5, and MPK3/6 [38]. To this regard, higher expressions of two SPEECHLESS genes in Jinhuang leaves suggest this genotype has ability for stomatal development better than Tainung 1 and Guiremang No. 82 under cold stress [39]. Moreover, the upregulation and/or exclusive expression of protein lin-54, sodium/hydrogen exchanger (NHX), calcium sensing receptor, malate transporter 12-like, and PATTERNING FACTOR-like protein 9 in Jinhuang leaves also indicate better stomatal operations under cold stress in Jinhuang than Guiremang No. 82 and Tainung 1. protein lin-54 homologs have been characterized for vascular patterning in Arabidopsis [40]. Whereas upregulation of calcium sensing indicate possible stomatal closure in Jinhuang but not in other genotypes based on its known function in Arabidopsis [41]. Taken together, our results propose that one of the key mechanisms through which Jinhuang has better cold stress tolerance than Tainung 1 and Guiremang No. 82 is MAPK signaling cascade involving Ca^2+^ and ROS related expression changes.

Phytohormones also play essential roles in signal transduction during cold stress. Particularly, JA, ethylene, ABA, BRs, and auxins have been implicated in cold stress responses [42]. The observation that relatively higher number of BR signaling-related genes were differentially expressed after cold stress is consistent with the roles of BRs i.e., cell elongation and division [43]. As noted in our results, CYCD3s and TCH4s were downregulated in Tainung 1 and Guiremang No. 82 compared to Jinhuang, indicating that the cell division and elongation under cold stress is halted or downregulated in Guiremang No. 82 and Tainung 1, while it continues in Jinhuang. Moreover, the observations related to photosynthesis and antenna proteins related gene expression together with BR signaling related changes is consistent with the fact that BRs positively regulate photoprotection during chilling stress [44]. The reduced expression of several PYLs, SnRK2s, and ABFs together with reducing ABA accumulation trend in three mango genotypes’ leaves suggest that cold stress responses are ABA independent [45]. There is also possibility that the increasing downregulation of several HOS3 transcripts in three mango varieties could be inhibiting ABA mediated stress responses as reported earlier [46]. This is an important observation and should be explored in detail in future studies for clear understanding. Similar to ABA, the reducing JA trends were interesting in three genotypes. This could be due to JA and ABA interactions as both hormones interact during abiotic stresses [47]. On the other hand, the higher and Jinhuang specific expressions of several MYCs could not only because of JA signaling but also by other phytohormones i.e., ethylene [48]. Though we didn’t detect ethylene related metabolites but the upregulation of ethylene regulators and many serine/threonine-protein kinase CTR1 transcripts (Table S5) is consistent with our proposed possibility of ethylene driven MYC2 expression changes. Finally, the cytokinins are also possibly regulating cold stress tolerance in mango leaves. We say this because the metabolome as well as transcriptome profiles showed differential changes after cold stress in all genotypes. The AHK upregulation sends signal to ARR-B, which has been shown to increased cold tolerance [49]. Taken together, the combined metabolome and transcriptome results indicate that cold stress reduces ABA and JA contents in leaves but increase cytokinin.

The ICE-CBF-COR signaling cascade enables plants to respond to cold stress. The differential expression of ICE-CBF-COR and related genes in three genotypes indicate that mango leaves also use this signaling cascade for cold stress responses. The limited knowledge on this signaling cascade in mango leaf further increases the importance of our results. The ICE1 regulates CBF/DREB1 expression. The upregulation of most CBF/DREB1 in three genotypes (except one in TLF) after 24 h of cold stress is indicative of cold perception and activation of ICE-CBF-COR signaling cascade. These genes trigger and regulate COR genes expression [50]. Since Jinhuang has been characterized as cold tolerant variety, the upregulation of COR27 like (*LOC123214877*, log_2_ foldchange = 4.09) and three other COR413_1 transcripts in JLF and/or JMF corresponds to its cold tolerance ability. These results are consistent with the *GhCOR27* gene that is involved in cold stress tolerance in cotton [51]. The relatively increased downregulation of COR413_1 like and COR413_2 in Tainung 1 compared to Jinhuang and Guiremang No. 82 also corresponds to its cold susceptibility [4]. Thus, the differential cold tolerance in these varieties could be due to the activation/inactivation of different COR genes.

Flavonoids like other secondary metabolites are accumulated in plants in response to cold stress [52]. A study in Arabidopsis indicated that FLs are determinants of cold acclimation [53]. Our results that flavonoid content increased after cold stress is consistent and imply that mango leaves exhibit similar mechanism. Particularly, the higher FLs in Jinhuang leaves are due to the increased expression of several flavonoid biosynthesis genes i.e., CA4H, F3H, C3’H, HCT, CCoAMT, CHS, and DFRs similar to leaves of blood orange under cold stress [54] (Fig. 6). Similar to our observations, study on Kiwi fruit [55] as well as maize [56] also exhibited increased FLs in cold tolerant varieties. In addition to FLs, AADs also increase with cold stress as reported in *Brassica rapa* [57]. The AAD accumulation trend in studied varieties indicate that its common response. Particularly, the increased expression of GS [58], enolase [59], pyruvate kinase [60], and serine O-acetyltransferase [61] in J after cold stress is consistent with their role in cold stress tolerance. Together with FLs, terpenoids are also known to accumulate in plants after exposure to cold stress [52]. Terpenoids enable plants to avoid oxidative stress during abiotic stress [62]. Therefore, the increase in terpenoids and FLs in Jinhuang leaves after cold stress, compared to Guiremang No. 82 and Tainung 1 leaves, indicates their cold stress protective role in Jinhuang. Finally, the increasing accumulation trends of alkaloids in Jinhuang leaves affirm that this genotype has better secondary metabolite biosynthesis potential compared to Tainung 1 and Jinhuang, which is due to increased expression of several pyridine and isoquinoline alkaloid biosynthetic genes such as chalcone synthases and primary-amine oxidase. Chalcone synthase is not only involved in alkaloid biosynthesis but is also a part of FL biosynthesis. Its increased expression causes accumulation of these metabolites and can be induced based on phytohormone signaling [63]. Taken together, our results indicate that cold stress induces increased secondary metabolite biosynthesis in mango leaves. Particularly, the higher cold tolerance in J could be linked with the increased secondary metabolite accumulation.

## Conclusion

**Fig. 7:**
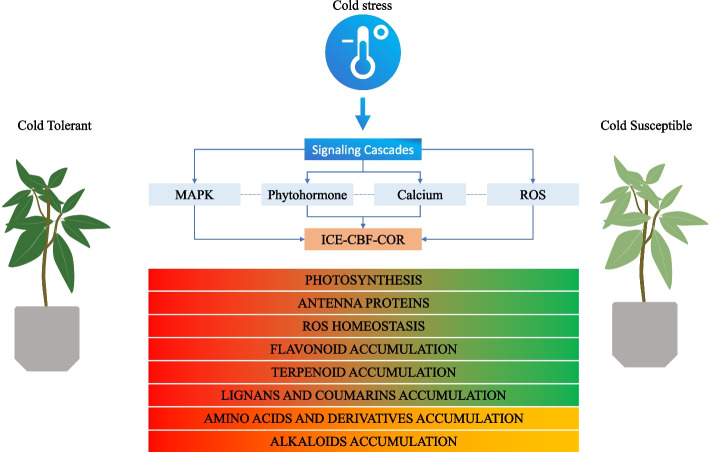
Cold stress response in mango leaves. Cold initiates signaling pathways and ICE-CBF-COR cascades to produce secondary metabolites in leaves. The bars with color gradient show higher (red) and lower (green) accumulation trends for each type of genotype. The bars with red and yellow-orange color indicate that the metabolites showed variable accumulation trends

In this work, metabolomic and transcriptomic analysis of cold stressed leaves of three mango cultivars were performed. Our results indicate that LC, terpenoids, FLs and tannins accumulate in higher amounts in the cold tolerant genotype compared to the other two genotypes. In general, AADs and NDs undergo large scale changes in mango leaves after cold stress treatment. The increased terpenoid content in cold tolerant genotype is due to several genes enriched in terpenoid backbone, diterpenoid and sesquiterpenoid biosynthesis pathway. Similarly, the increase in FL content in Jinhuang compared to Guiremang No. 82 and Tainung 1 leaves was consistent with increased expression of flavonoid biosynthesis genes. Among the phytohormones, JA and ABA showed decreased accumulation trends in all genotypes, but Jinhuang had relatively higher JA levels at all time points tested compared to other genotypes. Cytokinin levels increased with cold stress in most cases in three genotypes. We conclude that cold stress tolerance in mango leaves is associated with increased photosynthesis, accumulation of several secondary metabolite classes, interplay of phytohormones, MAPK and ICE-CBF-COR signaling cascades (Fig. 7). Our study provides several interesting observations and genes for future characterization and improvement of mango genotypes.

### Supplementary Information

**Supplementary Material 1. **

**Supplementary Material 2. **

**Supplementary Material 3. **

**Supplementary Material 4. **

**Supplementary Material 5. **

## Data Availability

The raw data has been submitted to NCBI SRA under the project number: PRJNA1014762 (https://www.ncbi.nlm.nih.gov/sra/PRJNA1014762).
