# Supplementary material for: Cold-stress induced metabolomic and transcriptomic changes in leaves of three mango varieties with different cold tolerance
Source: BMC Plant Biol. 2024 Apr 10;24:266. doi: 10.1186/s12870-024-04983-z (PMC11005188; doi:10.1186/s12870-024-04983-z)
Supplement: Supplementary file 1 — Supplementary Material 1. [file 12870_2024_4983_MOESM1_ESM.pptx]

## Slide 1
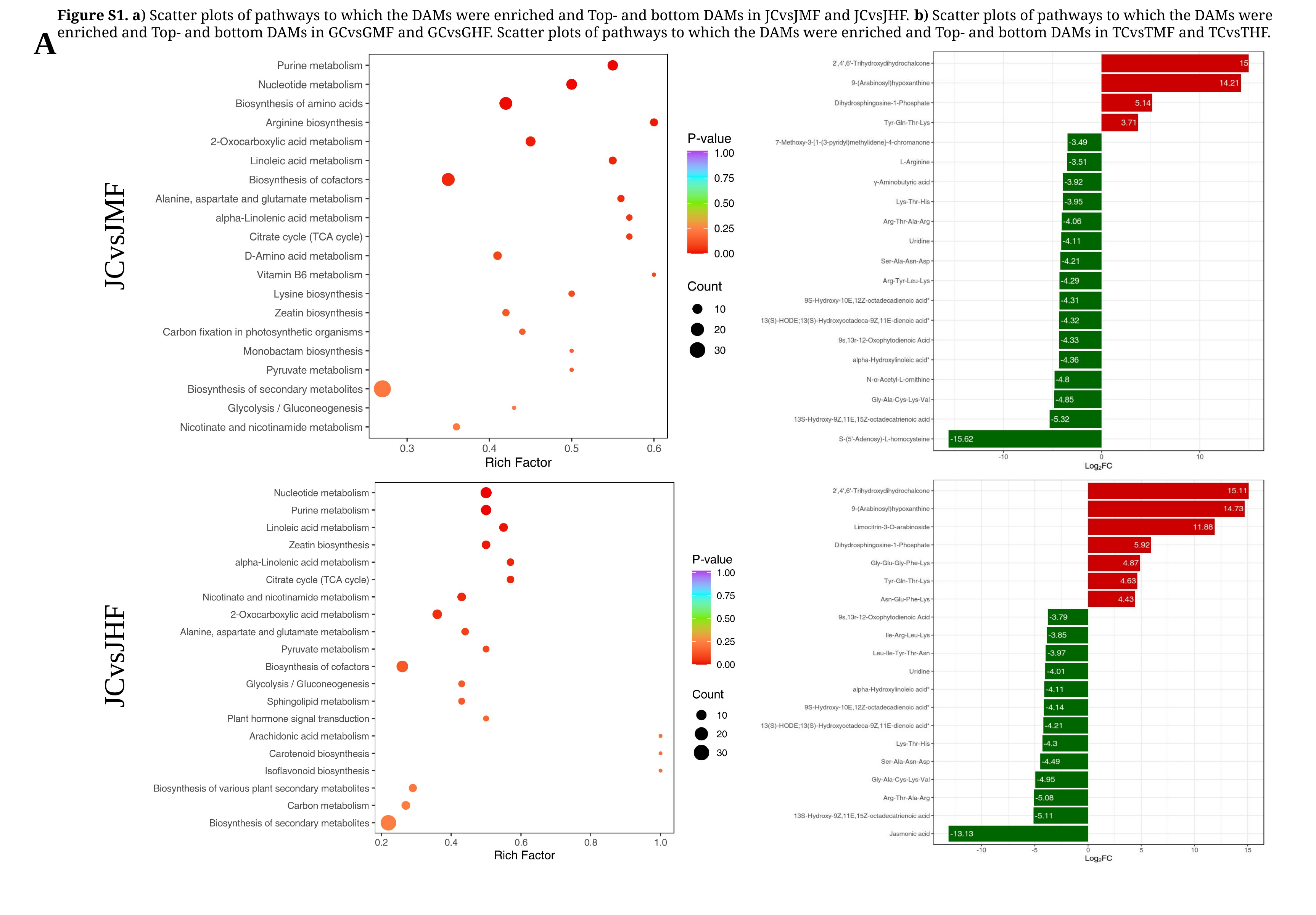

Figure S1. a) Scatter plots of pathways to which the DAMs were enriched and Top- and bottom DAMs in JCvsJMF and JCvsJHF. b) Scatter plots of pathways to which the DAMs were enriched and Top- and bottom DAMs in GCvsGMF and GCvsGHF. Scatter plots of pathways to which the DAMs were enriched and Top- and bottom DAMs in TCvsTMF and TCvsTHF.
A
JCvsJMF
JCvsJHF

## Slide 2
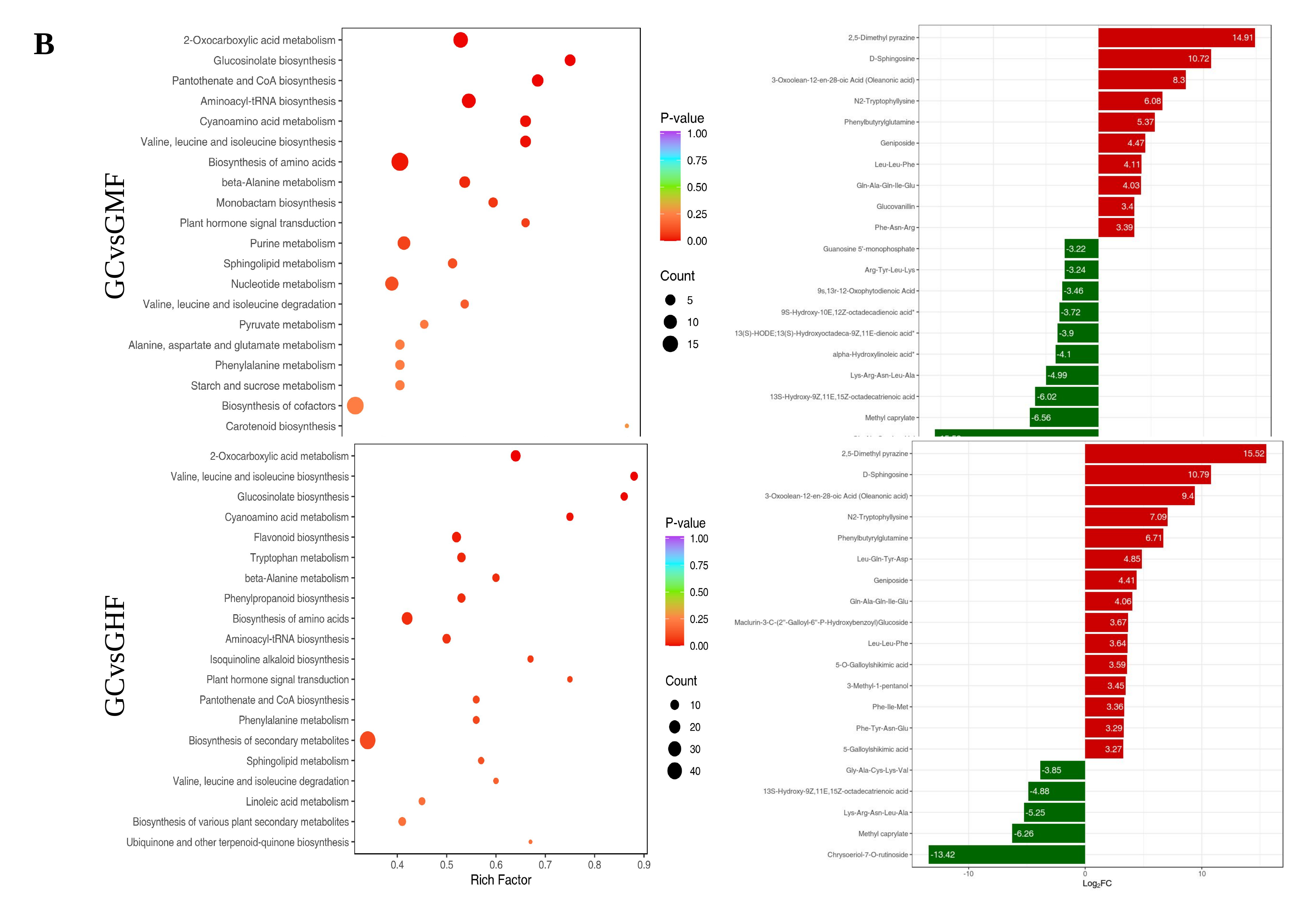

B
GCvsGMF
GCvsGHF

## Slide 3
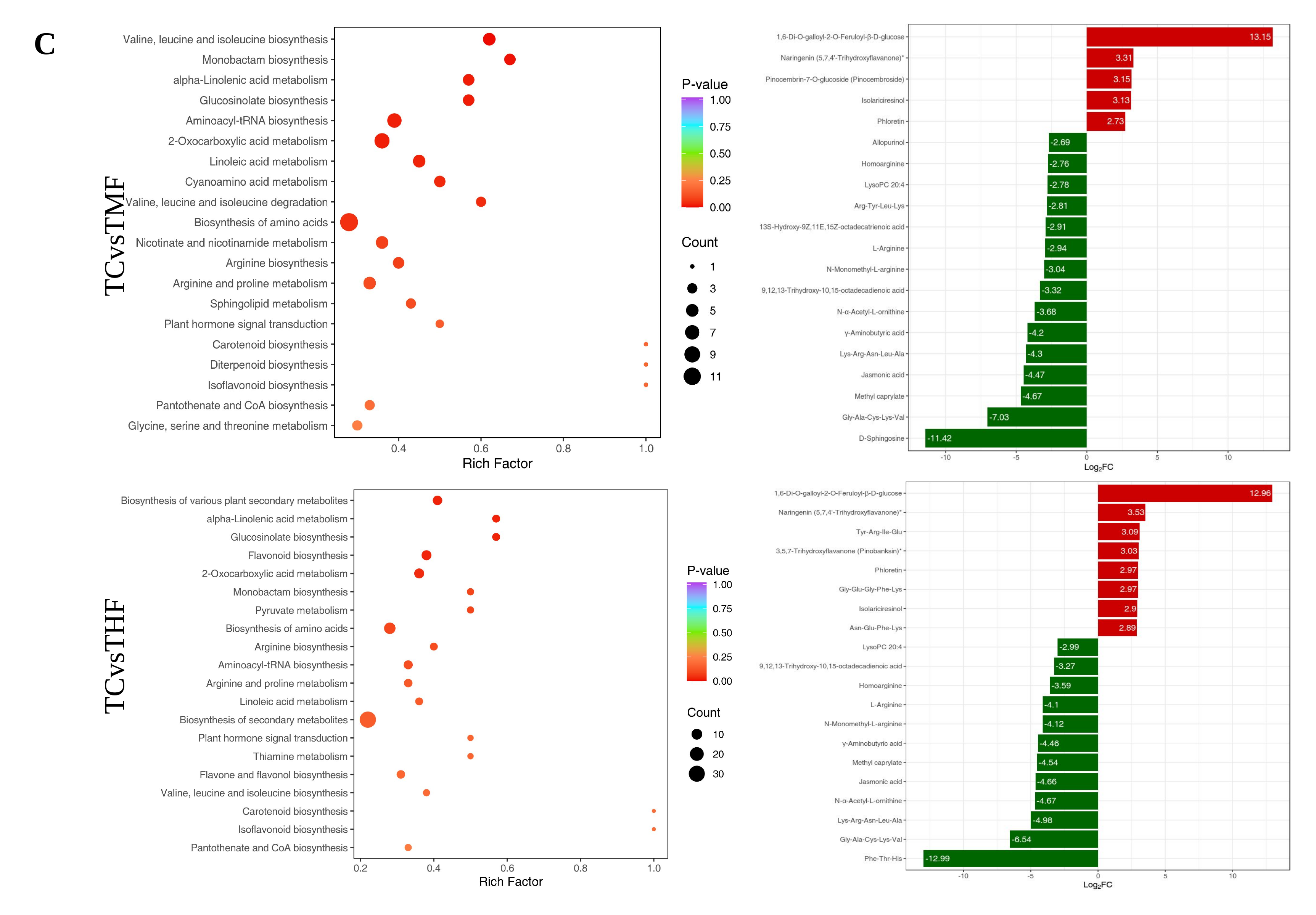

C
TCvsTMF
TCvsTHF
