# Supplementary material for: Cold-stress induced metabolomic and transcriptomic changes in leaves of three mango varieties with different cold tolerance
Source: BMC Plant Biol. 2024 Apr 10;24:266. doi: 10.1186/s12870-024-04983-z (PMC11005188; doi:10.1186/s12870-024-04983-z)
Supplement: Supplementary file 2 — Supplementary Material 2. [file 12870_2024_4983_MOESM2_ESM.pptx]

## Slide 1
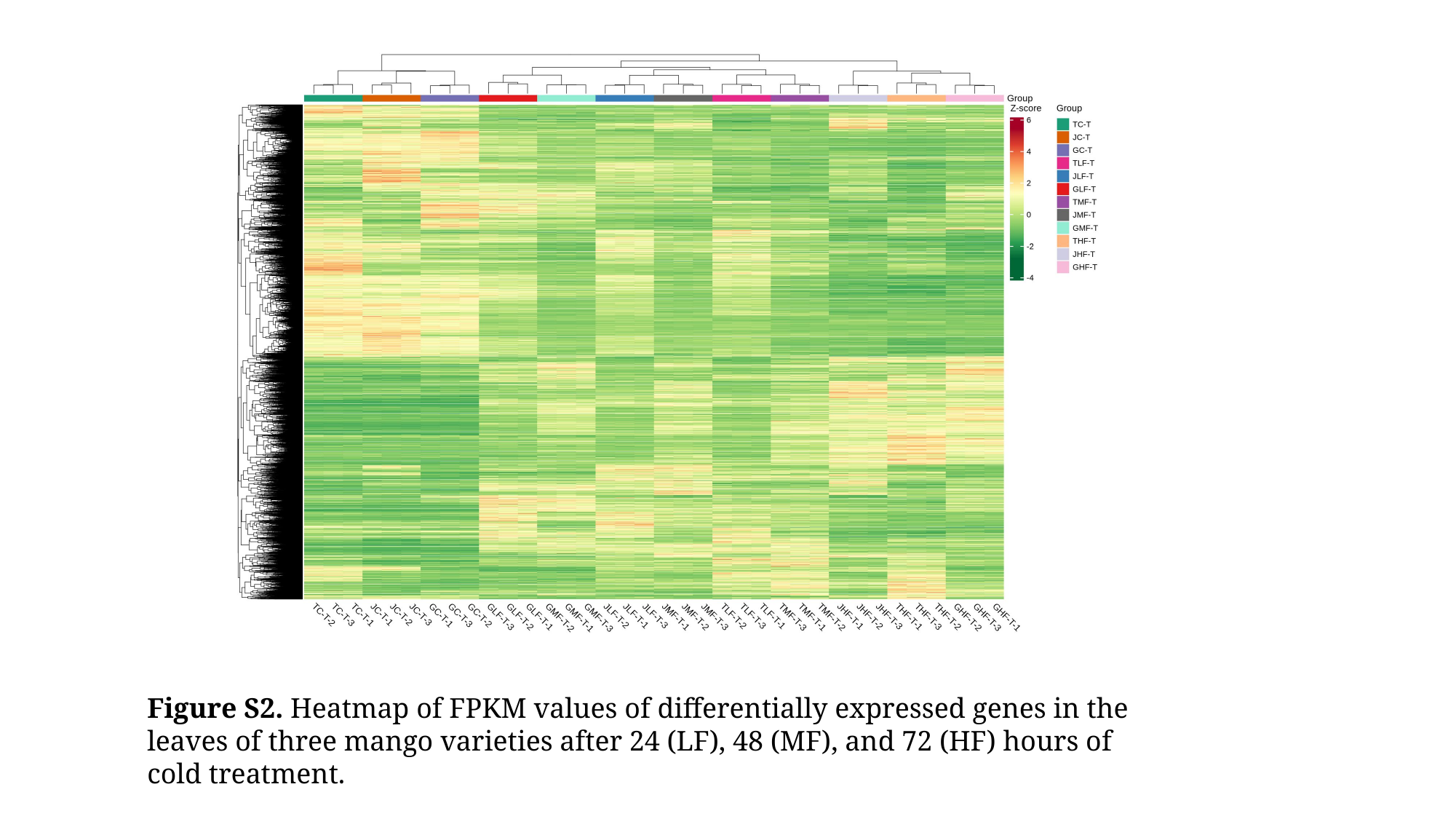

Figure S2. Heatmap of FPKM values of differentially expressed genes in the leaves of three mango varieties after 24 (LF), 48 (MF), and 72 (HF) hours of cold treatment.
