# Supplementary material for: Cold-stress induced metabolomic and transcriptomic changes in leaves of three mango varieties with different cold tolerance
Source: BMC Plant Biol. 2024 Apr 10;24:266. doi: 10.1186/s12870-024-04983-z (PMC11005188; doi:10.1186/s12870-024-04983-z)
Supplement: Supplementary file 3 — Supplementary Material 3. [file 12870_2024_4983_MOESM3_ESM.pptx]

## Slide 1
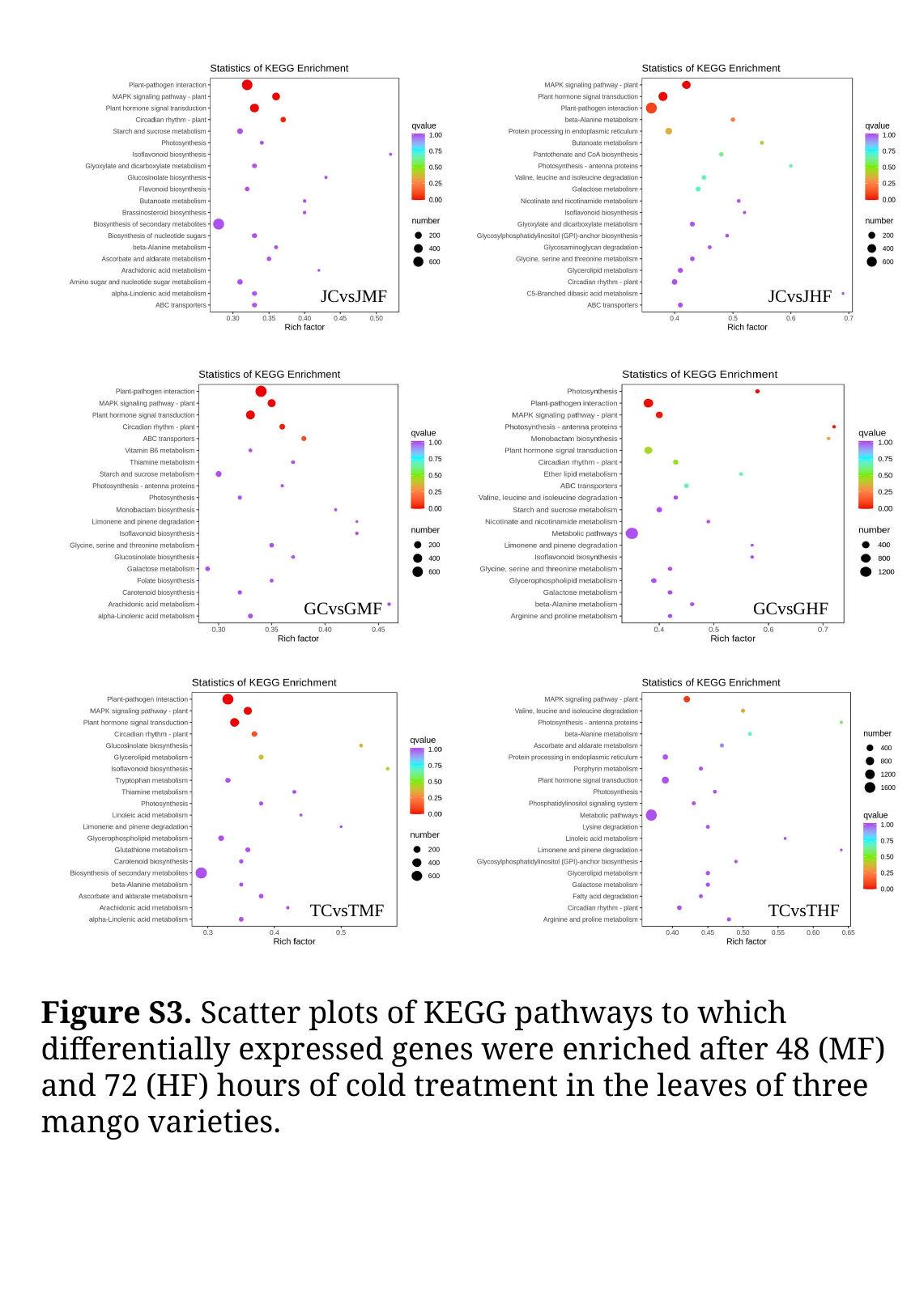

JCvsJHF
JCvsJMF
GCvsGHF
GCvsGMF
TCvsTMF
TCvsTHF
Figure S3. Scatter plots of KEGG pathways to which differentially expressed genes were enriched after 48 (MF) and 72 (HF) hours of cold treatment in the leaves of three mango varieties.
