# Supplementary material for: Cold-stress induced metabolomic and transcriptomic changes in leaves of three mango varieties with different cold tolerance
Source: BMC Plant Biol. 2024 Apr 10;24:266. doi: 10.1186/s12870-024-04983-z (PMC11005188; doi:10.1186/s12870-024-04983-z)
Supplement: Supplementary file 4 — Supplementary Material 4. [file 12870_2024_4983_MOESM4_ESM.pptx]

## Slide 1
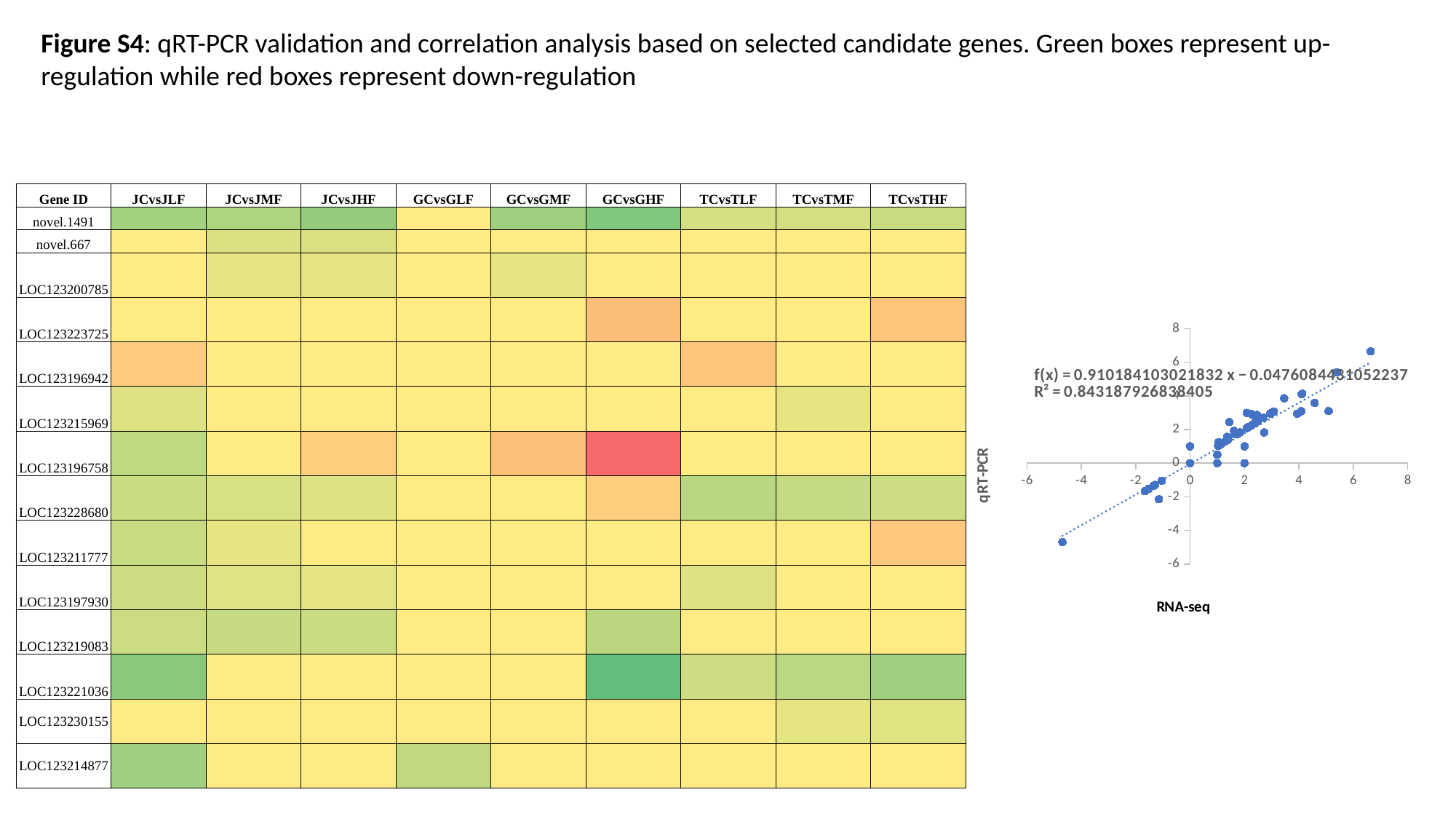

Figure S4: qRT-PCR validation and correlation analysis based on selected candidate genes. Green boxes represent up-regulation while red boxes represent down-regulation
| Gene ID | JCvsJLF | JCvsJMF | JCvsJHF | GCvsGLF | GCvsGMF | GCvsGHF | TCvsTLF | TCvsTMF | TCvsTHF |
| --- | --- | --- | --- | --- | --- | --- | --- | --- | --- |
| novel.1491 | | | | | | | | | |
| novel.667 | | | | | | | | | |
| LOC123200785 | | | | | | | | | |
| LOC123223725 | | | | | | | | | |
| LOC123196942 | | | | | | | | | |
| LOC123215969 | | | | | | | | | |
| LOC123196758 | | | | | | | | | |
| LOC123228680 | | | | | | | | | |
| LOC123211777 | | | | | | | | | |
| LOC123197930 | | | | | | | | | |
| LOC123219083 | | | | | | | | | |
| LOC123221036 | | | | | | | | | |
| LOC123230155 | | | | | | | | | |
| LOC123214877 | | | | | | | | | |
### Chart
| Category | RNA-seq |
|---|---|
